# Cholera in the Russian Sanatory Cordon

**Published:** 1847-06

**Authors:** 


*The Cholera in the Russian Sanatary Cordon.*—It is announced in the latest intelligence from Armenia, that the cholera has broken out in the sanatary cordon of Russian troops stationed on the borders of the Caspian Sea. Hence it has passed in a northwesterly direction to the Tartar districts of Solgan and Leokeran. Many of the Cossacks on the frontiers of Persia had perished. The whole of the western coast of the Caspian Sea, from Baku to Astrachan, is in a very unhealthy condition. The disease was still prevailing at Asterabad, Reschid, Ispahan, and Tiflis. All the inhabitants, who were able, have deserted the last mentioned town. Gastric affections were observed to be the forerunners of cholera, and those who were left in a weak state by these diseases, generally fell victims.—*Ib.*

